# Ocular biometry and refractive outcomes using two swept-source optical coherence tomography-based biometers with segmental or equivalent refractive indices

**DOI:** 10.1038/s41598-019-42968-3

**Published:** 2019-04-25

**Authors:** Miki Kamikawatoko Omoto, Hidemasa Torii, Sachiko Masui, Masahiko Ayaki, Kazuo Tsubota, Kazuno Negishi

**Affiliations:** Department of Ophthalmology, Keio University School of Medicine, Tokyo, Japan

**Keywords:** Refractive errors, Refractive errors, Refractive errors, Outcomes research, Outcomes research

## Abstract

This study compared the axial length (AL), central corneal thickness (CCT), anterior chamber depth (ACD), lens thickness (LT), mean anterior corneal radius of curvature (Rm), and postoperative refractive outcomes obtained from two different swept-source optical coherence biometers, the ARGOS (Movu, Nagoya, Japan), which uses the segmental refractive index for each segment, and the IOLMaster 700 (Carl Zeiss Meditec, Jena, Germany), which uses an equivalent refractive index for the entire eye. One hundred and six eyes of 106 patients with cataracts were included. The refractive outcomes using the Barrett Universal II, Haigis, Hoffer Q, and SRK/T formulas were evaluated. The mean AL, CCT, ACD, and Rm differed significantly (P < 0.001) with the IOLMaster 700 (25.22 mm, 559 µm, 3.23 mm, and 7.69 mm) compared with the ARGOS (25.14 mm, 533 µm, 3.33 mm, and 7.66 mm). The mean LTs did not differ significantly. The percentages of eyes within ±0.50 and ±1.00 diopter of the predicted refraction did not differ significantly (P > 0.05). The accuracy of the intraocular lens power calculations was clinically acceptable with both biometers, although the ocular biometry using these two biometers exhibited certain differences.

## Introduction

Precise measurement of the ocular parameters is essential to satisfy the demands for accurate postoperative refraction when implanting intraocular lenses (IOLs) during cataract surgeries. Previous studies have reported that every 1.0 mm of erroneous measurements of the corneal radius, axial length (AL), and anterior chamber depth (ACD) can result in 5.7, 2.7, and 1.5 diopters (D) of refractive error, respectively^1^. Olsen reported that the contributions to error from the ACD, AL, and corneal power were 42%, 36%, and 22%, respectively^1^.

In 1986 and 1988, Fercher *et al*. first used partial coherence interferometry (PCI) to measure the AL^2,3^, and Carl Zeiss (Jena, Germany) commercially launched optical biometry based on this technology in 1999 with the introduction of the IOLMaster^4^, which subsequently became the gold standard for measuring biometric data and IOL power calculations. Several devices are available for clinical use with different measurement systems such as PCI and optical low coherence reflectometry (OLCR), including the Lenstar LS900 (Haag-Streit, Verkauf, Switzerland)^5^, Aladdin (Topcon, Tokyo, Japan)^6^, AL-Scan (Nidek Co., Aichi, Japan)^7,8^, Galilei G6 (Ziemer, Port, Switzerland)^9,10^, OA-2000 (Tomey, Nagoya, Japan)^11,12^, and Pentacam AXL (Oculus, Wetzlar, Germany)^13–15^.

Optical biometry is now popular among cataract surgeons because it can obtain non-contact measurements and has replaced immersion ultrasound biometry in many settings. Recently, an optical biometer using swept-source optical coherence topography (SS-OCT), i.e., the IOLMaster 700 (Carl Zeiss Meditec, Jena, Germany), now is used extensively. The repeatability and reproducibility of the instrument are high and agree well with the parameters measured by PCI or a time-domain OCT technology biometer^16–18^. According to the manufacturer, this instrument calculates the AL using an equivalent refractive index, and the data is corrected by the undisclosed method in anatomically nonstandard eyes.

A new SS-OCT biometer, the ARGOS (Movu, Aichi, Japan), introduced in 2015, is unique in that it uses a segmental refractive index for each segmental length when calculating the AL. The measurement data from the ARGOS were reported to be comparable to that of biometers using PCI or OLCR with a high AL acquisition rate^19,20^. Theoretically, the AL data calculated using segmental refractive indices for each segment is more precise than the one using a single equivalent refractive index, and it is especially applicable for nonstandard anatomical eyes since the ratio of each part is not uniform. For example, an AL of a long eye usually consists of anatomically averaged anterior segment length and long vitreous cavity length^21^, which may result in an apparently shorter AL when measured using a single equivalent refractive index because the refractive index of vitreous is lower than the equivalent refractive index.

However, to our best knowledge, no studies have compared the measurement data and postoperative refractive outcomes obtained using the ARGOS using segmental refractive indices with those of other SS-OCT biometers using a single equivalent refractive index.

The purpose of the current study was to compare the measurement data and the postoperative refractive outcomes using two swept-source optical coherence tomography-based biometers with segmental indices and a single equivalent refractive index.

## Results

The study sample was comprised of 106 eyes (80 right eyes) of 106 patients (48 men, 58 women; mean age, 67.0 ± 9.9 years; range, 43–91 years); three eyes were excluded because the corneal diameters of these eyes were outside the 8- to 14-mm range and the predicted refraction could not be calculated using the Barrett Universal II formula with the ARGOS. In this sample, the mean power of the implanted IOLs was 17.9 ± 4.9 D (range, 6.0–26.5 D).

Table 1 shows all parameters obtained from the two optical biometers. Significant (P < 0.05) differences were seen in the AL, central corneal thickness (CCT), ACD, and mean anterior corneal radius of curvature (Rm) measurements in the entire AL range and in the medium and long ALs.

**Table 1:** Comparison of biometric measurements using two optical biometers AL.

Parameter	Optical Biometer				P value
	ARGOS		IOLMaster700		
	Mean ± SD	Range	Mean ± SD	Range	
**Over entire AL range (n = 106)**					
AL (mm)	25.14 ± 1.90	22.24, 30.64	25.22 ± 1.95	22.15, 30.89	<0.001
CCT (μm)	533 ± 32	458, 624	559 ± 32	476, 653	<0.001
ACD (mm)	3.33 ± 0.42	2.18, 4.36	3.23 ± 0.42	2.06, 4.22	<0.001
LT (mm)	4.47 ± 0.44	3.47, 5.66	4.46 ± 0.43	3.48, 5.64	0.515
Rm (mm)	7.66 ± 0.28	6.93, 8.14	7.69 ± 0.28	6.94, 8.17	<0.001
**Medium AL group (n = 76)**					
AL (mm)	24.16 ± 1.08	22.24, 25.86	24.20 ± 1.11	22.15, 25.97	<0.001
CCT (μm)	532 ± 33	458, 624	557 ± 35	476, 653	<0.001
ACD (mm)	3.20 ± 0.38	2.18, 4.03	3.10 ± 0.38	2.06, 3.93	<0.05
LT (mm)	4.53 ± 0.43	3.47, 5.66	4.53 ± 0.42	3.48, 5.64	0.882
Rm (mm)	7.63 ± 0.27	6.93, 8.12	7.66 ± 0.26	6.94, 8.12	<0.05
**Long AL group (n = 30)**					
AL (mm)	27.64 ± 1.01	25.90, 30.64	27.79 ± 1.04	26.01, 30.89	<0.001
CCT (μm)	537 ± 29	470, 597	562 ± 26	522, 617	<0.001
ACD (mm)	3.65 ± 0.33	3.11, 4.36	3.56 ± 0.32	3.00, 422	<0.001
LT (mm)	4.30 ± 0.42	3.58, 5.24	4.29 ± 0.41	3.53, 5.24	0.246
Rm (mm)	7.75 ± 0.29	7.13, 8.14	7.77 ± 0.30	7.15, 8.17	<0.001

SD = standard deviation.

**Figure 1:**
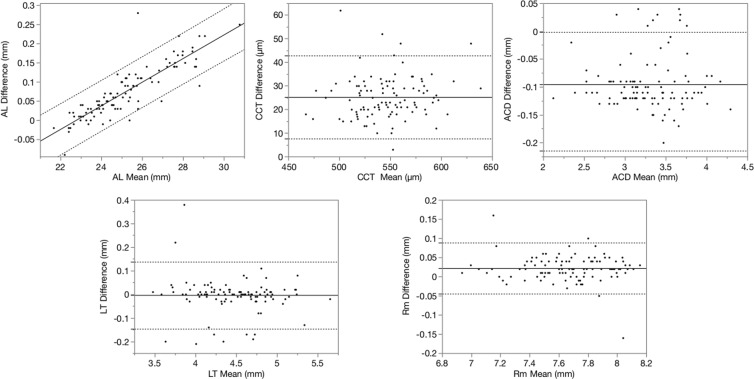
Bland-Altman plots show the agreements between the two biometers in the AL, CCT, ACD, LT, and Rm (n = 106). The solid lines indicate the mean difference. The dashed lines indicate the 95% LoAs.

Figure 1 shows the Bland-Altman plots for the agreement of various parameters between the two biometers. The mean differences were as follows: AL, +0.07 mm (P < 0.001; 95% limits of agreement [LoA], -0.06 to 0.21); CCT, +25 μm (P < 0.001; 95% LoA, 7 to 43); ACD, − 0.10 mm (P < 0.001; 95% LoA, − 0.19 to − 0.002); lens thickness (LT), − 0.004 mm (P = 0.515; 95% LoA, − 0.15 to 0.14); and Rm + 0.02 mm (P < 0.001; 95% LoA, − 0.04 to 0.09). The respective 95% LoAs for the AL, CCT, ACD, LT, and Rm were 0.27 mm, 35 μm, 0.19 mm, 0.29 mm, and 0.13 mm. Linear regression showed excellent correlations with the AL (r = 0.9999, r^2^ = 0.9997, P < 0.001), CCT (r = 0.9610, r^2^ = 0.9235, P < 0.001), ACD (r = 0.9936, r^2^ = 0.9872, P < 0.001), LT (r = 0.9861, r^2^ = 0.9725, P < 0.001), and Rm (r = 0.9925, r^2^ = 0.9852, P < 0.001).

Table 2 shows the percentages of eyes with arithmetic prediction errors less than ±0.50 and ±1.00 D for the entire AL range, medium-long, and long ALs. For the medium ALs, two formulas (Haigis and SRK/T) provided higher percentages of eyes with arithmetic prediction errors of ±0.50 D or less when the calculation was based on the measurements of the IOLMaster 700 compared with the ARGOS. For the long ALs, all formulas provided higher percentages of eyes with arithmetic prediction errors of ±0.50 D or less when the calculations were based on the measurements of the ARGOS compared with the IOLMaster 700, although the differences did not reach significance.

**Table 2:** Comparison of prediction errors for each formula using two devices.

Arithmetic prediction error within (%)	Formula	Optical Biometer		P value
		ARGOS	IOLMaster 700	
**Over entire AL range (n** = **106)**				
±0.50 D	Barrett Universal II	66.0	62.3	0.481
	Haigis	68.9	68.9	1
	Hoffer Q	58.5	56.6	0.815
	SRK/T	63.2	62.3	1
±1.00 D	Barrett Universal II	92.5	91.5	1
	Haigis	93.4	93.4	1
	Hoffer Q	92.5	88.7	0.289
	SRK/T	92.5	91.5	1
**Medium AL group (n** = **76)**				
±0.50 D	Barrett Universal II	64.5	61.8	0.791
	Haigis	67.1	71.1	0.607
	Hoffer Q	67.1	67.1	1
	SRK/T	61.8	68.4	0.277
±1.00 D	Barrett Universal II	92.1	90.8	1
	Haigis	93.4	93.4	1
	Hoffer Q	93.4	93.4	1
	SRK/T	92.1	89.5	0.625
**Long AL group (n** = **30)**				
±0.50 D	Barrett Universal II	70.0	63.3	0.625
	Haigis	73.3	63.3	0.250
	Hoffer Q	36.7	30.0	0.625
	SRK/T	66.7	46.7	0.070
±1.00 D	Barrett Universal II	93.3	93.3	1
	Haigis	93.3	93.3	1
	Hoffer Q	90.0	77.7	0.125
	SRK/T	93.3	96.7	1

**Figure 2:**
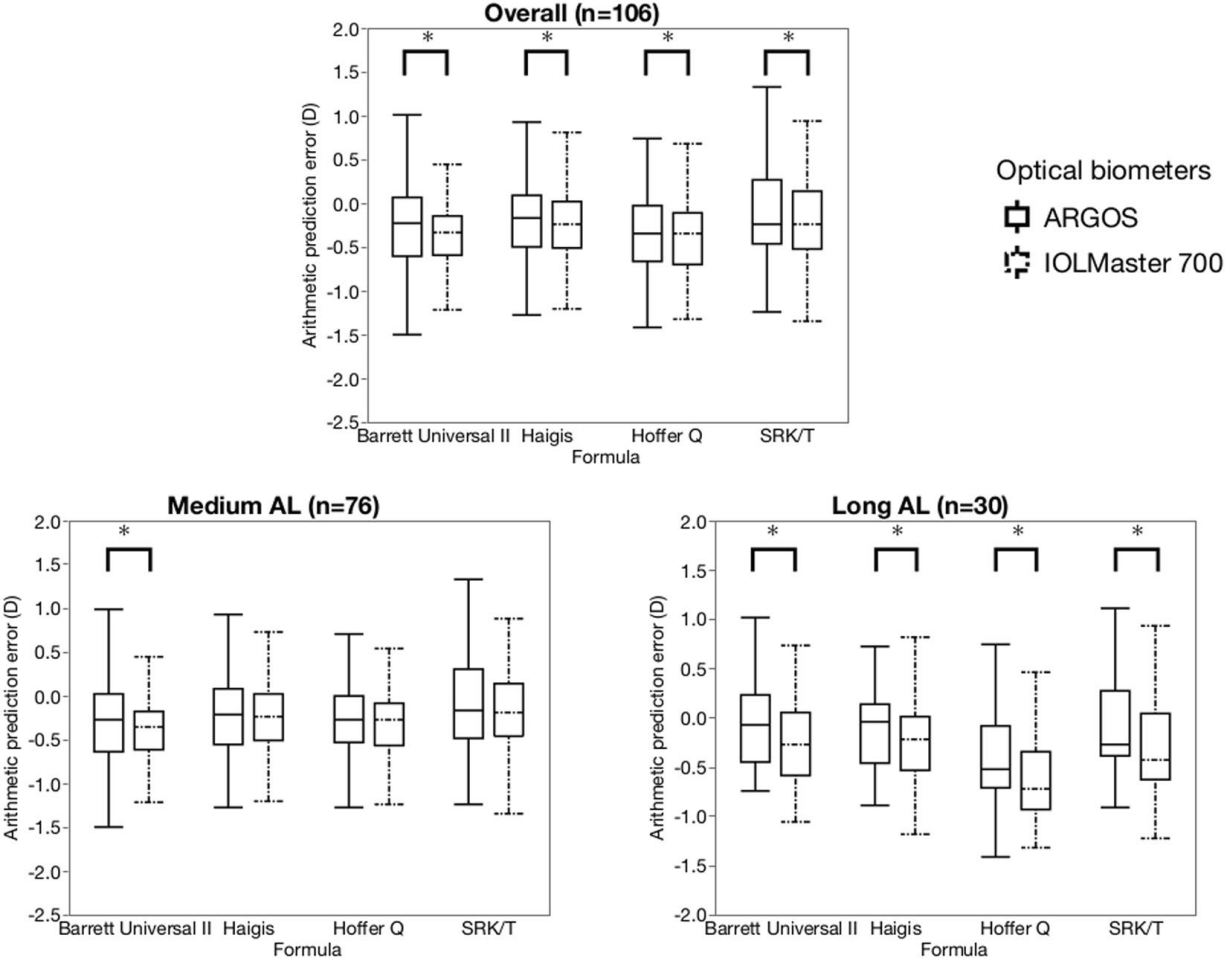
Distribution of the arithmetic prediction error in refraction with the four IOL power calculation formulas and the two optical biometers for the entire AL range, medium and long AL subgroups. *P < 0.05.

Figure 2 shows the distribution of the arithmetic prediction errors for both devices for the entire, medium, and long ALs. The overall median arithmetic prediction errors were closer to zero with the ARGOS than with the IOLMaster 700 for all four formulas (P < 0.001). For the medium ALs, the median arithmetic prediction errors also were closer to zero with the ARGOS than with the IOLMaster 700 using the Barrett Universal II formula (P < 0.001). For the long ALs, the arithmetic prediction errors were closer to zero with the ARGOS than with the IOLMaster 700 using all four formulas (P < 0.001).

**Figure 3:**
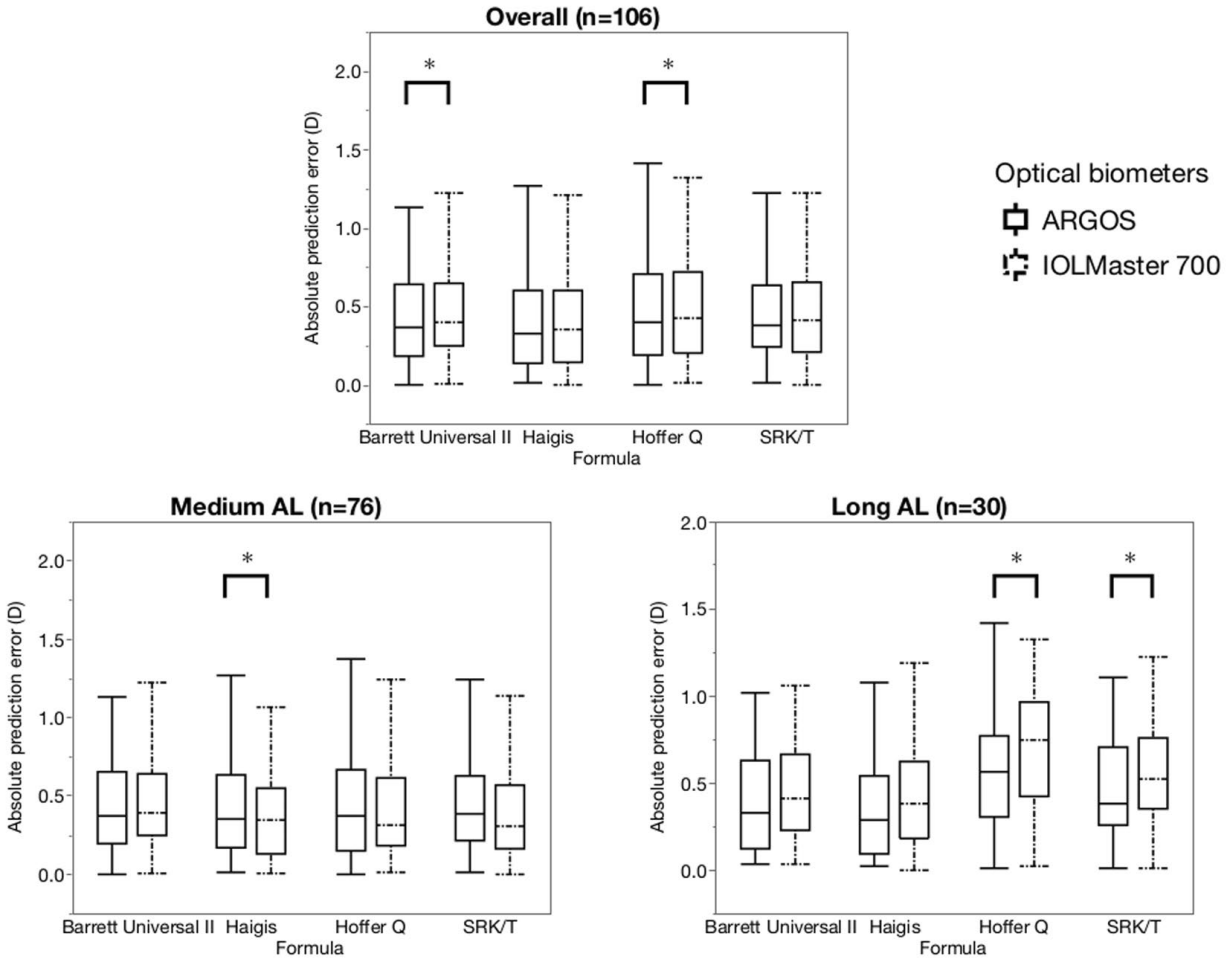
Distribution of the absolute prediction error in refraction with the four IOL power calculation formulas and the two optical biometers for the entire AL range, medium and long AL subgroups. *P < 0.05.

Figure 3 shows the distributions of the absolute prediction errors with each formula. For the medium ALs, the mean absolute errors (MAEs) with the IOLMaster 700 were significantly smaller than those of the ARGOS with the Haigis formula. For the long ALs, the MAEs of the ARGOS were significantly smaller than that of the IOLMaster 700 with the Hoffer Q and SRK/T formulas.

## Discussion

To the best of our knowledge, this is the first study to compare ocular biometry and postoperative refractive outcomes between two different SS-OCT-based optical biometers, one of which used the equivalent refractive index of the entire eye and the other the individual refractive index of each segment.

Comparisons of the AL, CCT, and Rm showed that the parameters obtained using the IOLMaster 700 were longer than those of the ARGOS, while the ACD obtained using the ARGOS was longer than that of the IOLMaster 700. One study reported agreement between the AL measurements of the ARGOS and IOLMaster (version 5)^20^, which is a PCI biometer. Higashiyama *et al*.^20^ reported that the AL obtained with the ARGOS was significantly shorter than that of the IOLMaster when measuring the long ALs, and their findings agreed with the current results.

Regarding the AL measurements, there was excellent agreement between the AL measurements obtained using the two SS-OCT units. The SS-OCT signal wavelengths in both optical biometers capture the tear layer and the retinal pigment epithelium (RPE) layer. The IOLMaster 700 converts that measurement into the length between the tear layer and the inner limiting membrane. In fact, the Zeiss IOLMaster series simulates precise segmental immersion ultrasound measurements by means of its built-in conversion relations^22^, but the conversion formula is disclosed. The ARGOS defines the foveal retinal thickness as 300 μm and subtracts 300 μm from the measurement between the tear layer and RPE layer. The ARGOS also uses a segmental refractive index for each segmental length when calculating the AL, and the IOLMaster 700 uses an equivalent refractive index; however, their specific numerical values have not been disclosed. A previous study reported that the AL difference between adults was due mainly to the difference in the vitreous cavity^21^. The ARGOS calculates segmental parameters by dividing the length measured in the refractive index as 1 by the segmental refractive index. Khokhar *et al*.^23^ reported that the ACD and CCT did not lengthen based on the AL. Therefore, the longer the AL becomes, the longer the posterior vitreous body becomes, and the ARGOS defines the vitreous refractive index as 1.336. Therefore, it is possible that the equivalent refractive index used in the IOLMaster 700 exceeds 1.336. Faria-Ribeiro *et al*.^24^ reported that the assumption of a single equivalent refractive index of 1.3549 used by the IOLMaster PCI biometer is optimized for an AL value near 24 mm with a LT around 3.6 mm. Yang *et al*.^25^ reported that the fixation status and presence of posterior staphyloma are important factors in AL measurements in myopic eyes, neither of which was investigated in the current study.

There was good agreement between the ACD measurements obtained from the two instruments. The ACD obtained using the ARGOS was longer than that provided by the IOLMaster 700. Jeong *et al*.^26^ reported that the preoperative ACD has the greatest effect in the IOL power calculations of the third-generation formulas and Haigis formula. The ACD measurement also is essential for determining the indication for phakic IOL implantation. The difference in the data should be kept in mind in clinical use.

There was good agreement between the Rm readings obtained from the two units. A difference in the Rm was seen between the two devices. Both define the keratometric index as 1.3375 and use light-emitting diode (LED) projection for keratometry. However, the ARGOS measures the K reading using a 2.2-mm optical diameter, and the IOLMaster 700 uses a 2.5-mm optical diameter. The difference in the measurement area possibly caused the steeper keratometric value obtained by the ARGOS.

There was only moderate agreement between the CCT measurements obtained using the two devices. We observed a considerable 30-μm difference in the CCT between the ARGOS and IOLMaster 700. In the current study, the CCT obtained using the IOLMaster 700 was thicker than reported previously^16–18^, while the CCT obtained using the ARGOS was similar to that reported previously^19^. Both biometers measure the CCT in the optical axis and the anterior corneal position is recognized as the surface tear layer. We also compared the data using these two devices with the data measured by the anterior-segment OCT (SS-1000, Tomey, Japan) and found that the mean CCT with the anterior-segment OCT was between the mean CCTs measured using the ARGOS and the IOLMaster 700, and the CCT with the anterior-segment OCT was differed significantly from both of the data obtained using the two devices (data not shown). The differences in the refractive index, the design (e.g., the detection threshold of signal wave), and ocular surface condition (e.g., the defects of the lacrimal layer caused by dry eye and opening deficiency of an eyelids) at the time of measurements might have resulted in the difference in the CCTs between the two biometers. The reason for this difference requires further investigation.

In the Bland-Altman plots, a few cases were not within the 95% LoA. But in most cases, the difference between the two devices was considered clinically insignificant. Seven of 10 samples, which were not within the 95% LoA in the ACD measurement, also were not within the 95% LoA in the LT measurement. In these samples, the deeper the ACD was, the thinner the LT was. We confirmed that there were no problems in the measurement conditions, such as a fixation displacement or an error in the signal wavelength. The cause of this discrepancy is unknown; however, the detection error of the borderline between the anterior chamber and the anterior lens surface might have resulted in the measurement errors.

Preoperatively, IOL powers have been calculated using the Barrett Universal II^27^, Haigis^22^, Hoffer Q^28^, and SRK/T formulas^29^. The AL and keratometry are used to calculate the IOL power in the SRK/T and Hoffer Q formulas, and the AL, keratometry, and ACD in the Barrett Universal II and Haigis formulas.

The comparison of the arithmetic prediction errors between the ARGOS and IOLMaster 700 indicated that both biometers showed a myopic trend, and the median arithmetic prediction errors were closer to zero with the ARGOS than with the IOLMaster 700; the longer the AL became, this trend became more marked, suggesting that the ARGOS showed a significant hyperopic trend compared with the IOLMaster 700, especially with long ALs. A reason for this outcome is the fact that the AL and Rm of the IOLMaster 700 were longer than those of the ARGOS, while the ACD of the ARGOS was longer than that of the IOLMaster 700. In the current study, the short AL group included a small number of eyes and no comparison was performed. A previous study reported that the AL measured with the ARGOS was longer than that with the IOLMaster (version 5)^20^ in the short AL group (AL, <23.27 mm). Therefore, it is expected that among eyes with short ALs, the ARGOS will exhibit a more myopic trend than the IOLMaster 700, which requires further study.

No significant differences were seen in the percentage of eyes within the prediction errors of ±0.50 and ±1.00 D. However, the comparison of the two devices showed that the ARGOS provided a lower absolute error with the Hoffer Q and SRK/T formulas in eyes with long ALs. Among these eyes, the current formulas tended to choose IOLs with insufficient powers, leaving patients with postoperative hyperopia^30–32^. The ARGOS showed a significantly more hyperopic trend than the IOLMaster 700 and provided smaller median absolute prediction errors with all four formulas and lower absolute prediction errors with the Hoffer Q and SRK/T formulas in eyes with long ALs. This might be the advantage of using a segmental refractive index instead of an equivalent refractive index, especially in eyes with long ALs. In contrast, the IOLMaster 700 provided lower absolute errors with the Haigis formula in the eyes with medium ALs. In this case, the optimized IOL constants for the Zeiss IOLMaster were used with both optical biometers. Therefore, the ARGOS may provide lower absolute prediction errors in the eyes with medium and medium long ALs when the IOL constant is optimized for the ARGOS.

The current study had some limitations. First, the study sample included neither short eyes (AL < 22.0 mm) nor very long eyes (AL > 32.0 mm). Second, we did not consider nuclear sclerosis. The ARGOS defines the segmental refractive index of the lens as 1.410; however, the refractive index of the lens changes depending on nuclear sclerosis. This factor requires further study. Finally, we did not conduct repeatability tests, although the repeatability values of both the ARGOS^19^ and the IOLMaster 700^16–18^ measurements were excellent.

In conclusion, the parameters obtained from the ARGOS and IOLMaster 700 differed significantly. The refractive outcomes using the ARGOS were comparable to those of the IOLMaster 700, both of which were clinically acceptable. The refractive outcomes using segmental refractive indices (i.e. the ARGOS) showed a significant hyperopic trend and less arithmetic prediction errors compared with those using equivalent refractive index (i.e. the IOLMaster 700), especially in eyes with long ALs.

## Methods

### Patients and surgery

This study was a retrospective chart review of patients who underwent cataract surgery (uncomplicated phacoemulsification and aspiration and IOL implantation) performed between July 2016 and July 2018 at Keio University Hospital. Only the right eye was included in this study if a patient underwent bilateral cataract surgeries. The exclusion criteria were eyes with a history of a previous corneal or intraocular surgery, any corneal disease, and postoperative best-corrected visual acuity below 0.8 (20/25) for any reason. All patients provided written informed consent before cataract surgery, and opt-out consent was used to participate in this study. The institutional review board of Keio University approved the study, which was conducted in accordance with the principles of the Declaration of Helsinki. One surgeon (K.N.) performed all surgeries through a temporal near-clear 2.2-mm incision under topical anesthesia.

### Instrumentation

We examined all participants on the same day using both the ARGOS and IOLMaster 700. The AL, CCT, ACD, LT, and Rm were recorded and the keratometric index of refraction was set at 1.3375 for both biometers.

ARGOS is an optical biometer that uses SS-OCT with a 1,060-mm laser infrared light to generate OCT images of the complete ocular length, but it differs from the IOLMaster 700 in the use of a segmental refractive index for each segmental length when calculating the AL. The ARGOS is designed to measure the AL, keratometry, CCT, ACD, LT, horizontal white-to-white (WTW) corneal diameter, and pupillary size (PS). An automatic algorithm evaluates all biometry parameters, and the optical distances are converted into geometric distances using the standard refractive indices of 1.376 for the cornea, 1.336 for the aqueous and vitreous, and 1.410 for the lens. The ARGOS defines the AL as the distance between the cornea and the internal limiting membrane of the retina and the retinal thickness as 300 μm. The ARGOS also provides OCT images of the complete longitudinal section of the eye, which can be used to check the fixation status. The keratometry is obtained from OCT information in combination with a ring LED with a laser wavelength of 860 nm. The K reading was measured using 16 projected lights from the LED ring at the 2.2-mm diameter.

The IOLMaster 700 also uses SS-OCT technology with a laser wavelength of 1,060 nm to generate optical B-scans (optical cross-sections) to measure the ocular biometric data. The speed of the length measurement system allows acquisition of full-eye length tomograms at 2,000 A-scans/second. It also acquires the AL, keratometry, CCT, ACD, LT, WTW, and PS. The WTW is measured using the LED light source. The IOLMaster provides OCT images of the complete longitudinal section of the eye. The AL measurement is the average value of three scans in each of six meridians. The IOLMaster uses telecentric keratometry with a 950-nm LED light source, making it distance-independent, and OCT imaging to detect abnormal lens geometries. The K readings are calculated by analyzing the anterior corneal curvature at 18 reference points in hexagonal patterns at about the 1.5-, 2.5-, and 3.5-mm optical zones, and the final average K readings are calculated^33^. The K reading analyzed at the 2.5-mm optical zone was used for the IOL power calculations in accordance with the previous model.

### IOL power calculation and constant optimization

Preoperatively, the IOL powers were calculated using the Barrett Universal II^27^, Haigis^22^, Hoffer Q^28^, and SRK/T formulas^29^. A final evaluation was performed by assessing the subjective spherical equivalent refractive outcomes 1 month postoperatively. To calculate the prediction errors in the refraction, the predicted postoperative refraction based on the IOL power actually implanted according to each formula was subtracted from the measured spherical equivalent refraction. Thus, a positive prediction error indicates a refractive outcome that was more hyperopic than predicted. The mean arithmetic prediction error, MAE, median absolute error, and the percentages of eye with arithmetic prediction errors within ± 0.50 D and ± 1.00 D^34^ were calculated. The Tecnis ZCB00 and ZCB00V IOLs (J & J Vision, New Brunswick, NJ) were used as the models for IOL calculations and the User Group for Laser Interference Biometry online table (Available at: http://www.augenklinik.uni-wuerzburg.de/ulib/c1.htm. Accessed March 30, 2018). The optimized IOL constants for the Zeiss IOLMaster were used with both optical biometers (optimized IOL constant A = 119.3 for the SRK/T formula; a0 = − 1.302, a1 = 0.210, and a2 = 0.251 for the Haigis formula; personalized ACD = 5.80 for Hoffer Q formula; and the recommended constant of 119.3 for the Barrett Universal II formula), because IOL constants specialized for the ARGOS were available at present, and the manufacturer recommended their use.

Subgroup analysis was performed for eyes with medium ALs (22.00 ≤ AL < 26.00 mm) and long ALs (AL ≥ 26.00 mm) according to the ALs measured with the IOLMaster 700.

### Statistical analysis

Statistical analysis was performed using the SPSS for Microsoft software (V.25, IBM Corporation, Armonk, NY, USA) and the JMP Pro version 14 (SAS Institute, Inc., Cary, NC, USA) under the supervision of a statistician. The Kolmogorov-Smirnov test was used to check the normal data distribution. A paired *t*-test and Wilcoxon test were used to compare the AL, ACD, CCT, LT, and keratometry values in the subsamples of patients in which the measurements were obtained using the two optical biometers. The correlations between the two instruments were assessed using the Pearson correlation coefficient. Agreements between the measurements using the two devices were calculated using Bland-Altman plots, and the 95% LoAs of all parameters were recorded. The differences between the measurements obtained with the two devices were plotted against their means^35^.

Because of the non-normal distribution of the arithmetic prediction errors and the absolute prediction errors, the Friedman test with Bonferroni’s correction was used to compare the absolute prediction errors with the four IOL power calculation formulas in each subgroup for each device. The Wilcoxon signed-rank test was used to compare the difference errors of each formula with the two biometers. The difference in the absolute prediction errors among the formulas was assessed using the Friedman test. The percentages of eyes with arithmetic prediction errors within ±0.50 and ±1.00 D were compared using the McNemar test. Statistical significance was set at P < 0.05.
