# Author Correction: Ocular biometry and refractive outcomes using two swept-source optical coherence tomography-based biometers with segmental or equivalent refractive indices

**DOI:** 10.1038/s41598-020-69871-6

**Published:** 2020-07-31

**Authors:** Miki Kamikawatoko Omoto, Hidemasa Torii, Sachiko Masui, Masahiko Ayaki, Kazuo Tsubota, Kazuno Negishi

**Affiliations:** Department of Ophthalmology, Keio University School of Medicine, Tokyo, Japan

Correction to: *Scientific Reports*https://doi.org/10.1038/s41598-019-42968-3, published online 25 April 2019

The version of this Article previously published quoted an incorrect email address for Prof Kazuno Negishi. Correspondence and requests for materials should also be addressed to kazunonegishi@keio.jp

The original version of this Article contained a typographical error in the Abstract.

“This study compared the axial length (AL), central corneal thickness (CCT), anterior chamber depth (ACD), lens thickness (LT), mean anterior corneal radius of curvature (Rm), and postoperative refractive outcomes obtained from two different swept-source optical coherence biometers, the ARGOS (Movu, Nagoya, Japan), which uses the segmental refractive index for each segment, and the IOLMaster 700 (Carl Zeiss Meditec, Jena, Germany), which uses an equivalent refractive index for the entire eye. One hundred and six eyes of 106 patients with cataracts were included.”

now reads:

“This study compared the axial length (AL), central corneal thickness (CCT), anterior chamber depth (ACD), lens thickness (LT), mean anterior corneal radius of curvature (Rm), and postoperative refractive outcomes obtained from two different swept-source optical coherence biometers, the ARGOS (Movu, Aichi, Japan), which uses the segmental refractive index for each segment, and the IOLMaster 700 (Carl Zeiss Meditec, Jena, Germany), which uses an equivalent refractive index for the entire eye. One hundred and six eyes of 106 patients with cataracts were included.”

Additionally, there was a typographical error in the Introduction section where,

“Several devices are available for clinical use with different measurement systems such as PCI and optical low coherence reflectometry (OLCR), including the Lenstar LS900 (Haag-Streit, Verkauf, Switzerland)^5^, Aladdin (Topcon, Tokyo, Japan)^6^, AL-Scan (Nidek Co., Aichi, Japan)^7,8^, Galilei G6 (Ziemer, Port, Switzerland)^9,10^, OA-2000 (Tomey, Nagoya, Japan)^11,12^, and Pentacam AXL (Oculus, Wetzlar, Germany)^13,14,15^.”

now reads:

“Several devices are available for clinical use with different measurement systems such as PCI and optical low coherence reflectometry (OLCR), including the Lenstar LS900 (Haag-Streit, Verkauf, Switzerland)^5^, Aladdin (Topcon, Tokyo, Japan)^6^, AL-Scan (Nidek Co., Aichi, Japan)^7,8^, Galilei G6 (Ziemer, Port, Switzerland)^9,10^, OA-2000 (Tomey, Aichi, Japan)^11,12^, and Pentacam AXL (Oculus, Wetzlar, Germany)^13,14,15^.”

Also there was a typographical error in the Introduction section where,

“Recently, an optical biometer using swept-source optical coherence topography (SS-OCT), i.e., the IOLMaster 700 (Carl Zeiss Meditec, Jena, Germany), now is used extensively.”

now reads:

“Recently, an optical biometer using swept-source optical coherence tomography (SS-OCT), i.e., the IOLMaster 700 (Carl Zeiss Meditec, Jena, Germany), now is used extensively.”

This Article also contained an error in the title of Table 1 where,

“Comparison of biometric measurements using two optical biometers AL.”

now reads:

“Comparison of biometric measurements using two optical biometers.”

Additionally, this Article contained a typographical in the Discussion section where,

“In fact, the Zeiss IOLMaster series simulates precise segmental immersion ultrasound measurements by means of its built-in conversion relations^22^, but the conversion formula is disclosed.”

now reads:

“In fact, the Zeiss IOLMaster series simulates precise segmental immersion ultrasound measurements by means of its built-in conversion relation^22^, but the conversion formula is undisclosed.”

These errors have now been corrected in the HTML and PDF versions of the Article.

